# Supplementary material for: The Prevalence of Inappropriate Image Duplication in Biomedical Research Publications
Source: mBio. 2016 Jun 7;7(3):e00809-16. doi: 10.1128/mBio.00809-16 (PMC4941872; doi:10.1128/mBio.00809-16)
Supplement: Data Set S1 — , DOCX file, 0.1MB [file mbo003162846sd1.docx]

Supplementary Data S1: R code

Duplicated Figures R stats - Elisabeth Bik - Stanford University - May 2016

========================================================

# Analysis of a set of 782 papers with problematic images, identified in a search of 20,621 papers published in 40 journals.

# Libraries and initialization:

```{r}

R.Version()

library("ggplot2")

packageVersion("ggplot2")

library("ape")

packageVersion("ape")

library("vegan")

packageVersion("vegan")

library(scales)

packageVersion("scales")

theme_set(theme_bw())

```

# Choose your workspace (change to your own environment)

setwd("/Users/EliesBik/Desktop/") #home

#### PART 1: Figures 1 and 5: Plotting number of screened papers per year and percentage of papers found per year

# Note that we used Excel-generated versions of these figures in the paper, not these versions, but the data is the same.

```{r}

years <- read.table("YearsForR.txt", header=TRUE)

head(years)

class(years)

dim(years)

# Plotting number of screened papers per year, different colors for Journal 1 and other journals

ggplot(years) + geom_histogram(aes(x=Year, y=ScreenedAll), stat="identity", fill="grey", color="black") + geom_histogram(aes(x=Year, y=Screened39), stat="identity", fill="black") + scale_x_continuous(breaks = pretty_breaks(n=20)) + theme(axis.text.x = element_text(angle = 45, vjust = 1, hjust=1))

# Plotting number of problematic papers per year

ggplot(years) + geom_histogram(aes(x=Year, y=PercPPAll), stat="identity", fill="grey", color="black") + scale_x_continuous(breaks = pretty_breaks(n=20)) + theme(axis.text.x = element_text(angle = 45, vjust = 1, hjust=1))

```

#### PART 2: Exploring relationship between percentage of problematic papers and impact factors of 40 journals (Lines 177-180) (Figure 6)

# Here we will only consider the 17816 papers screened in the last 10 years; 738 of these contained problems.

```{r}

# Import table with relationship between % problematic papers (PPs) and impact factors

journals <- read.table("JournalsImpactFactorsForR.txt", header=TRUE)

head(journals)

class(journals)

dim(journals)

# plotting impact factor vs % pps as an x,y scatter plot, linear scale

# Using ggplot function

# To plot 11 categories use color scheme Spectral, Set3, Paired

# the label= parts are needed to force linear model to use the whole set, not split up by aesthetics

# Here we group papers from Cell, Nature, and Science under the same publisher, otherwise we run out of colors

IF <-journals$ImpactFactor

PP <- journals$ProblemPapers

plot <- ggplot(journals, aes(IF, PP)) + geom_point(size=4, aes(color=Publisher2)) + scale_color_brewer(palette="Paired")

plot

# trying a linear model (using a log formula) and a Loess regression on the plot

plot + stat_smooth(method = "loess") # Exploring shape of best fit with a localized regression model

plot + stat_smooth(method = "lm") # applying a linear regression model on the non-logarithmic plot - not a very good fit.

plot + stat_smooth(method = "lm", formula = y~log(x)) # log-based regression model appears to be best fit

# make the x axis logarithmic to better display the dense datapoint concentration at lower impact factors

# define tickmarks manually

# library(scales) was needed for tick marks

# removed the minor grid lines which are not useful in log displays

logplot <- plot + scale_x_log10(breaks = c(1,2,3,4,5,6,7,8,9,10,20,30,40,50),limits=c(1, 50)) + theme(panel.grid.minor = element_blank())

logplot

# adding a trendline to the log plot, linear regression model

# The grey zone is the 95% confidence interval

# The loess plot explores localized best fit but was not used in paper

# see also http://www.ats.ucla.edu/stat/r/faq/smooths.htm

logplot + stat_smooth(method = "loess")

logplot + stat_smooth(method = "lm")

# Testing correlation between impact factor and percentage of problematic papers

# Using basic R - tested both Pearson and Spearman

x <- journals$ImpactFactor

y <- journals$ProblemPapers

cor.test(x,y, method="pearson")

# Output of Pearson's product-moment correlation

# data: x and y

# t = -2.4496, df = 38, p-value = 0.01902

# alternative hypothesis: true correlation is not equal to 0

# 95 percent confidence interval:

# -0.61055724 -0.06528413

# sample estimates: cor -0.3692835

cor.test(x,y, method="spearman")

# Output of Spearman's rank correlation rho

# data: x and y

# S = 15079.53, p-value = 0.0005791

# alternative hypothesis: true rho is not equal to 0

# sample estimates: rho -0.5262678

```

#### Testing problem paper ratios between Open Access (oa) journals vs non open access (noa) journals (Lines 180-182)

# We screened 40 journals, 12 of which were open access

# Here we will only consider data obtained from the last 10 years; total of 17816 papers screened, with 738 problem papers found.

```{r}

oa <- c(456,10262)

noa <- c(282,6816)

data.table = rbind(oa,noa)

data.table

chisq.test(data.table)

# output: Pearson's Chi-squared test with Yates' continuity correction

# data: data.table

# X-squared = 0.7832, df = 1, p-value = 0.3762

# conclusion: no significant difference in percentage of problem papers between open access vs non-open access

```

#### PART 3: Country of affiliation vs percentage of problematic images (Lines 190-196; Figure 7)

```{r}

# Country of Affiliation vs problematic paper percentages

# This data derived from 348 problematic papers found among 8138 papers in Journal 1 (PLOS ONE)

# For this analysis the countries of affiliation was considered; a paper could be associated with more than one country.

affs <- read.table("CountriesForR.txt", header=TRUE)

head(affs)

dim(affs)

# Plotting the data

# Affiliations (x axis) are the affiliations of all 8138 papers.

# Problems (y axis) are the affilations of the 348 problem papers. We expected this relationship to be linear.

affplot <- ggplot(affs, aes(Published, Problems, label=Country)) + geom_point(size=4, color="red")

affplot + geom_abline(intercept=0,slope=1) + geom_text(hjust=-0.1,vjust=-0.1)

# Plotting the data on a logarithmic scale to better bring out the journals in the crowded bottom left part of the plot

affplot + scale_x_log10(breaks = c(0.01,0.02,0.03,0.04,0.05,0.06,0.07,0.08,0.09,0.1,0.2,0.3,0.4,0.5),limits=c(0.01, 0.5)) + scale_y_log10(breaks = c(0.01,0.02,0.03,0.04,0.05,0.06,0.07,0.08,0.09,0.1,0.2,0.3,0.4,0.5),limits=c(0.01, 0.5)) + theme(panel.grid.minor = element_blank()) + theme(axis.text.x = element_text(angle=90, vjust=0.5)) + geom_abline(intercept=0.002,slope=1, color="blue") + geom_text(hjust=-0.1,vjust=-0.1,angle=45)

# Chi square test for country of screened papers vs country of problem papers

# Can input these numbers manually

# 8138 papers from Journal 1 screened; 347 had problems

# Columns are PP vs non-PP

# Rows are Yes Country, No Country (e.g. China)

# Papers affiliated with China:

prim <- c(172,1958)

sec <- c(176,5832)

data.table = rbind(prim,sec)

data.table

chisq.test(data.table)

# Pearson's Chi-squared test with Yates' continuity correction

# data: data.table

# X-squared = 100.4648, df = 1, p-value < 2.2e-16

# Papers affiliated with USA:

prim <- c(85,3246)

sec <- c(263,4544)

data.table = rbind(prim,sec)

data.table

chisq.test(data.table)

# Output: Pearson's Chi-squared test with Yates' continuity correction

# data: data.table

# X-squared = 40.2572, df = 1, p-value = 2.226e-10

```

#### PART 4: Chi square test for recidivist percentage (Lines 205-214)

# primary data set: we found 782 problem papers among 20,621 screened papers (so 19839 had no problems)

# secondary data set (papers from authors on problem papers found in primary set): we found 269 problem papers among 2425 screened papers (so 2156 without problems)

# Can input these numbers manually

```{r}

prim <- c(782,19839)

sec <- c(269,2156)

data.table = rbind(prim,sec)

data.table

chisq.test(data.table)

# output: Pearson's Chi-squared test with Yates' continuity correction

# data: data.table

# X-squared = 264.0302, df = 1, p-value < 2.2e-16

```

#### THIS PART DOES NOT NEED TO BE INCLUDED

#### Most of this data is a bit older and the numbers are slightly different

# The following code was exploratory but not used in the paper

# Analysis of all 782 papers from 40 journals

```{r}

# Import simplified table with the 782 papers found in Journals 1-40

# the last command forces the ordering; otherwise the categories will be displayed in alphabetical order

papers2 <- read.table("All781Papers.txt", header=TRUE)

class(papers2)

head(papers2)

dim(papers2)

papers2$Problem = factor(papers2$Problem, c("Duplication", "Shift", "Alteration"))

papers2$Journal = factor(papers2$Journal, c("OtherJournals", "Journal1"))

# Plot the number of papers with 3 types of error: duplications, shifts, manipulations.

# Shifts appear to be the most common.

# The last plot switches the order of the legend, and changes the colors.

ggplot(papers2, aes(Problem)) + geom_bar(fill="grey", color="black")

ggplot(papers2, aes(Problem, fill=Journal)) + geom_bar()

ggplot(papers2, aes(Problem, fill=Journal)) + geom_bar() + scale_fill_manual(values=c("#666666", "#CCCCCC"), breaks=c("Journal1", "OtherJournals"))

ggplot(papers2, aes(Year, fill=Problem)) + geom_bar() + scale_fill_manual(values=c("#666666", "#CCCCCC","#CC3300" ))

# Plot the 3 types of error as stacked bar plot, split per country (main affiliation)

ggplot(papers2, aes(Country, fill=Problem)) + geom_bar() + theme(axis.text.x = element_text(angle=90, vjust=0.5)) + scale_fill_manual(values=c("#009933", "#FFCC66", "#CC3300"), breaks=c("Alteration", "Shift", "Duplication"))

# Plot the number of papers with 3 types of error, faceted per (first) country

ggplot(papers2, aes(Problem)) + geom_bar() + facet_wrap(~Country)

```

#### PART 3: Analysis of 347 problematic papers found among 8138 papers in Journal 1. Not all of these analyses were included in the paper

```{r}

# Import simplified table with the 347 papers found in Journal 1

# the last command forces the ordering; otherwise the categories will be displayed in alphabetical order

# Column "Country" is the affiliation of the first author, "Countries" list other affiliations. Both columns were counted.

papers <- read.table("J1PapersForR.txt", header=TRUE)

class(papers)

head(papers)

dim(papers)

papers$Problem = factor(papers$Problem, c("Dups", "Shifts", "Manips"))

# Plot the number of papers with 3 types of error: duplications, shifts, manipulations.

# Shifts appear to be the most common.

ggplot(papers, aes(Problem)) + geom_bar()

# Plot the 3 types of error as stacked bar plot, split per country (main affiliation)

ggplot(papers, aes(Country, fill=Problem)) + geom_bar() + theme(axis.text.x = element_text(angle=90, vjust=0.5)) + scale_fill_manual(values=c("#009933", "#FFCC66", "#CC3300"), breaks=c("Manips", "Shifts", "Dups"))

# Plot the number of papers with 3 types of error, faceted per (first) country

ggplot(papers, aes(Problem)) + geom_bar() + facet_wrap(~Country)

# Plot bar graph displaying the 3 types of error, and whether additional problem papers by same author(s) were found

ggplot(papers, aes(Problem, fill=FoundYN)) + geom_bar()

```

#### Testing problem paper ratios between Open Access (oa) journals vs non open access (noa) journals

# We screened 39 journals, 12 of which were open access

# Here we test all 20 years, which is not a good test because underrepresentation of OA papers in older years, so we did not include this in the paper

# The better test (only including data from last 10 years) is described above.

```{r}

oa <- c(456,10341)

noa <- c(325,9321)

data.table = rbind(oa,noa)

data.table

chisq.test(data.table)

# output: Pearson's Chi-squared test with Yates' continuity correction

# data: data.table

# X-squared = 0.2627, df = 1, p-value = 0.6083

# conclusion: no significant difference in percentage of problem papers between open access vs non-open access

```
